# The Multitasking Potential of Alarmins and Atypical Chemokines

**DOI:** 10.3389/fmed.2019.00003

**Published:** 2019-01-23

**Authors:** Aphrodite Kapurniotu, Ozgun Gokce, Jürgen Bernhagen

**Affiliations:** ^1^Division of Peptide Biochemistry, Technische Universität München, Freising, Germany; ^2^System Neuroscience Laboratory, Institute for Stroke and Dementia Research, Klinikum der Universität München, Munich, Germany; ^3^Department of Vascular Biology, Institute for Stroke and Dementia Research, Klinikum der Universität München, Ludwig-Maximilians-University, Munich, Germany; ^4^Munich Heart Alliance, Munich, Germany; ^5^Munich Cluster for Systems Neurology (SyNergy), Munich, Germany

**Keywords:** alarmin, chemokine, cytokine, moonlighting, promiscuity, inflammation, cardiovascular disease, MIF protein family

## Abstract

When the human genome was sequenced, it came as a surprise that it contains “only” 21,306 protein-coding genes. However, complexity and diversity are multiplied by alternative splicing, non-protein-coding transcripts, or post-translational modifications (PTMs) on proteome level. Here, we discuss how the multi-tasking potential of proteins can substantially enhance the complexity of the proteome further, while at the same time offering mechanisms for the fine-regulation of cell responses. Discoveries over the past two decades have led to the identification of “surprising” and previously unrecognized functionalities of long known cytokines, inflammatory mediators, and intracellular proteins that have established novel molecular networks in physiology, inflammation, and cardiovascular disease. In this mini-review, we focus on alarmins and atypical chemokines such as high-mobility group box protein-1 (HMGB-1) and macrophage migration-inhibitory factor (MIF)-type proteins that are prototypical examples of these classes, featuring a remarkable multitasking potential that allows for an elaborate fine-tuning of molecular networks in the extra- and intracellular space that may eventually give rise to novel “task”-based precision medicine intervention strategies.

## Introduction

The human genome project was concluded in the beginning of this millennium (1). It came as a surprise that the genome only contains 21,306 protein-coding genes; this appeared to contradict the remarkable complexity of higher organisms. However, it turned out that complexity and diversity are substantially enhanced by other mechanisms such as alternative splicing, non-protein-coding transcripts, or post-translational modifications (PTMs) on proteome level. For example, protein translation and mRNA stability are regulated and fine-tuned by thousands of non-coding RNAs including microRNAs, small interfering RNAs (siRNAs), and long non-coding RNAs (lncRNAs). The complexity in the available battery of gene products in humans is further multiplied by alternative promoter sites and transcript splicing. Moreover, the size of the proteome is much larger than the predicted 21,306 proteins. It is amplified by a factor of >50-fold by a multitude of PTMs that determine the activity, interaction specificity, and sub-cellular localization of many cellular proteins (Table 1). Not all of these diversity-enhancing mechanisms operate at the same time and on the same protein class, but they certainly lead to a significant expansion of the proteome. While it is well-known that some PTMs alter the interaction profile of a protein and switch-on or switch-off certain protein-protein or protein-substrate binding events, interactions that are subject to PTM-modulated changes are typically confined to binding partners of the same or similar functional class. A prototypical example would be the regulation of the activity of the enzyme phosphofructokinase-2 (PFK-2), which, following post-translational phosphorylation, is converted from a kinase to a phosphatase, consistent with the inversion of substrate specificity from a mono-phosphorylated to a bis-phosphorylated sugar (i.e., the binding partner).

**Table 1:** The diversity in the human proteome is enlarged by multitasking proteins: atypical chemokines and MIF family proteins as role models.

**Genes/mechanism**	**Number/increase in diversity**	**Comment**
Protein-coding genes	21,306	^*^see recent publication
LncRNAs	18,484	Possibly up to 100,000
antisenseRNA	2,144	
MiscRNAs	1,228	
Splicing, alternative promoters	Amplification factor 6–8 x	
Post-translational Modifications Proteins including PTMs	Amplification factor >50 x >1,000,000	
**Amplification/Diversification by “multitasking” of ACKs/MIF proteins**		
MIF protein-coding genes	2	^**^a third one predicted
Topology-mediated diversity	Amplification factor 3 x	Extracellular vs. cytosolic vs. nuclear
Receptor-ligand promiscuity	Amplification factor 3–4 x	CD74, CXCR2, CXCR4, CXCR7
Receptor complexes	^*^Amplification factor 3–5 x	CD74/CXCR2, CD74/CXCR4, CXCR4/CXCR7
Multiple intracellular binding proteins	Amplification factor >5 x	CSN5/JAB1, Trx, Prx, mutSOD1, p53, BNPL1
Protein complex formation	^***^Amplification factor *3 x*	Homomeric/heteromeric as well as trimer-dimer-monomer equilibria
MIF proteins including PTMs	>5	MIF, MIF-2, SNO-MIF, proxMIF, oxMIF, glycoMIF^****^
Increase in MIF “functional” diversity depending on cell type, tissue, disease stage, expression stage etc.	Amplification factor 3 –100^***^ x	Collective increase (sum) over all possible diversity variants

* *Pertea et al. (2)*.

** the Uniprot databank lists a gene called DDTL that could be related to MIF and MIF-2;

*** estimated factor as the physiological relevance of MIF monomers or homomeric vs. heteromeric oligomers is unclear;

**** *in addition to unmodified MIF and MIF-2, according to some recent reports there are post-translationally modified variants of MIF (SNO-MIF, oxMIF, proxMIF, and GlcNAcMIF), on which cysteine residues, the N-terminal proline, or a C-terminal serine are modified. While possible functions of these oxidized and O-N-acetyl-glycosylated MIF species have been suggested, the exact mechanisms underlying such physiological and pathophysiological roles are currently incompletely understood (3–8)*.

Research in the last two decades has led to the discovery of numerous molecules that engage in unanticipated—second—activities, thus suggesting a novel concept of “molecular multitasking” or “molecular moonlighting.” The concept holds that certain biomolecules may execute functions in previously unexpected -different- locations, that they may utilize previously unrecognized -different- substrates, or that they may engage in previously unexpected -different- binding interactions, thus previously unexpected functions that often are seemingly unrelated to their established “main” function. The “molecular multitasking” or “molecular moonlighting” concept thus goes beyond the “one molecule, one function” paradigm. With respect to proteins it goes beyond the “one molecule, one-fold, one function” paradigm. The concept is appropriately described by the “moonlighting” definition which refers to the practice of holding a “second regular job” in addition to: one's main job” (https://www.collinsdictionary.com/de/worterbuch/englisch/moonlighting).

Alarmins and certain cytokines and chemokines are protagonistic examples of biomolecules executing “molecular multitasking/moonlighting.” In fact, work in the past two decades has led to the identification of surprising and previously unrecognized functionalities of long known cytokines, inflammatory mediators, and intracellular proteins, unraveling novel molecular networks in physiology, inflammation, and cardiovascular disease. To this end, alarmin research has established the idea that homeostatic intracellular proteins can have additional, non-homeostatic, extracellular functions that signal danger, and promote inflammation. While the alarmin category of molecules not only encompasses proteins, but also small molecule metabolites such as ATP or nucleic acids such as RNA, in this article, we will discuss only protein-type alarmins and atypical chemokines/cytokines with an emphasis on high-mobility group box-1 (HMGB-1) and macrophage migration-inhibitory factor (MIF)-type proteins. We will discuss their remarkable multitasking potential that enhances the diversity of the proteome and allows for a fine-tuning of molecular networks in the extra- and intracellular space in health and disease.

## Alarmins as Professional “Multitaskers”

Alarmins are molecules released from a damaged or diseased cell that upon release can stimulate a sterile immune or inflammatory response (9–12). Examples are heat-shock proteins such as HSP-70 or−90, intracellular cytokines such as interleukin-1 α (IL-1α) or IL-33, amino-acyl tRNA synthetase fragments or the p43 auxiliary component of the tRNA multisynthetase complex, chromatin proteins such HMGB1 or histones, whole nucleosomes, phagocyte inflammatory proteins such as the S100 proteins S100A8, S100A9, or S100A12, but also small molecule metabolites such as ATP or uric acid crystals. Alarmins are sometimes referred to as “endokines” to indicate that “endo”genous intracellular molecules acquire cyto'kine'-like functions once release into the extracellular space. There is a significant overlap between alarmins and the molecular class of danger-associated molecular patterns (DAMPs) or danger signals. The DAMP terminology was coined by symmetry to the concept of pathogen-associated molecular patterns (PAMPs), conserved pathogen-derived molecular motifs that are recognized by host pattern recognition receptors (PRRs), such as Toll-like receptors (TLRs) (the “stranger theory”). Accordingly, endogenous “molecular patterns” that signal cell or tissue damage and the need for the development of a sterile host inflammatory response were coined as DAMPs (the “danger theory”) (13). In fact, it has been suggested that alarmins and DAMPs are two terms for the same class of molecules and concept. We will preferentially use the term alarmin in this review article.

There are numerous excellent recent reviews on alarmins/DAMPs (9, 10, 13–18). Briefly, alarmins have a physiological, often homeostatic, role inside the cell, but take on additional, novel, functions when they are exposed to the extracellular environment. They signal “danger” to the host and trigger a local inflammatory response, which in physiologic healing leads over to tissue regeneration, but when dysregulated can cause pathologic inflammation and related disorders. Following tissue injury or cell stress, most alarmins are passively released from dead cells, but the release of some alarmins can also follow a controlled secretion program to signal early, sub-lethal cell stress. For protein-type alarmins, this regulated secretory process follows a so-called “unconventional” endoplasmic reticulum (ER)/Golgi-independent pathway (19).

**Figure 1:**
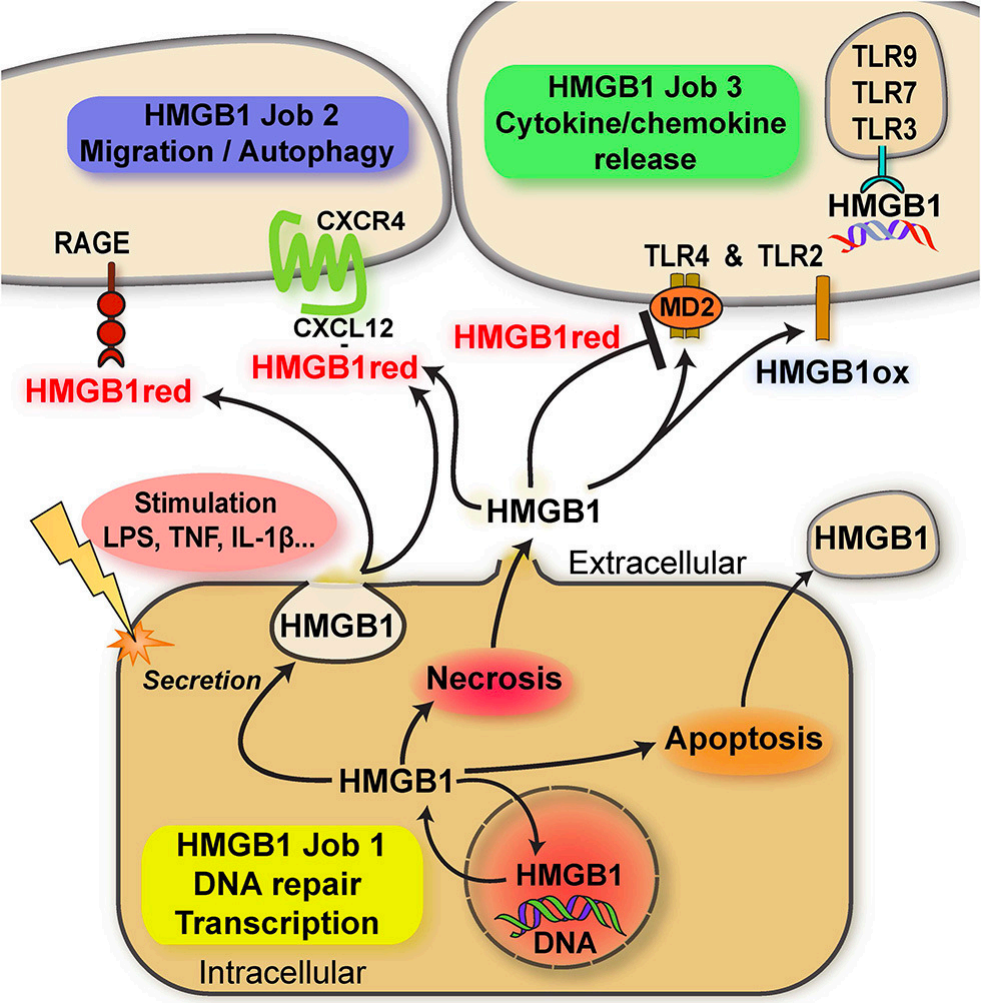
Scheme summarizing the multitasking/moonlighting functions of HMGB1. For details see manuscript text. HMGB1, high mobility group box-1; HMGB1red, fully reduced HMGB1; HMGB1ox, partially or fully oxidized HMGB1; TLR, Toll-like receptor; RAGE, receptor of advanced glycation endproducts (AGEs); MD2, myeloid differentiation factor 2/lymphocyte antigen 96.

Here, we focus on the “multitasking” concept as it applies to proteinaceous alarmins and their functional and molecular connection to atypical chemokines (ACKs) (for latter, see next chapter). This principle is best characterized by HMGB1, a typical “multitasking” alarmin and the first identified member of the HMGB family. HMGB1 is a ubiquitous nuclear DNA-binding protein that is highly conserved, present in most cell types, and that serves as nuclear/transcriptional cofactor (20). In addition to its intracellular nuclear function, HMGB1 can be released into the extracellular milieu following cell damage or via regulated secretion via an autophagolysosomal pathway (21). Extracellular HMGB1 fulfills a second independent function as an intercellular-acting cytokine and inflammatory mediator (10, 14, 17, 22–29). In analogy to the “multitasking”/“moonlighting” principle, transcriptional regulation by nuclear HMGB1 represents the physiological “day-time job” (job 1) inside the cell, while its “cytokine” activity outside the cell would be the additional “night-time” or “moonlighting” job (job 2) that HMGB1 takes on to signal cell stress or damage and initiate a rapid local inflammatory response (10, 14, 17) (Figure 1). Weighing the terms “multitasking” vs. “moonlighting,” the latter appears preferable as the definition of “multitasking” asks for the handling of multiple jobs at the same time. However, the “day-time” and “night-time” jobs of HMGB1 (and other “multitaskers”) are clearly separated in time and localization. Transcriptional coregulation by HMGB1 is restricted to the intracellular–nuclear-compartment and healthy homeostatic cell conditions, whereas the inflammation-promoting cytokine function of HMGB1 only occurs outside the cell. Inversely, nuclear HMGB1 is invisible to the host immune and tissue inflammatory system. The two HMGB1 “jobs” are thus distinctly separated in space and phase (Figure 1).

HMGB1 contains three conserved cysteines. These residues are redox-sensitive and their differential modification modulates the bioactivity of HMGB1 in the extracellular space via determining the binding specificity to three different receptors due to the induction of specific three-dimensional conformations. Disulfide formation between the C23 and C45 cysteines of HMGB1 and preservation of C106 in its reduced thiol form (“disulfide HMGB1”) enables for binding and signaling through the TLR4/MD-2 receptor axis. This activity leads to the induction of inflammatory cytokines in macrophages and may be termed the “cytokine task” of extracellular HMGB1 (22, 23, 30–32) (Figure 1). In contrast, the all-thiol conformer of HMGB1 may behave as a co-chemokine through heterocomplex formation with the classical CXC chemokine CXCL12. HMGB1/CXCL12 complexes bind to CXCR4 to promote chemotactic cell migration of monocytes/macrophages and T cells (33) (the “chemokine task”) (Figure 1). Alternatively, all-thiol HMGB1 binds to receptor of advanced glycation endproducts (RAGE) to trigger cell migration or autophagy (the “autophagy task”; Figure 1). Interestingly, RAGE and TLR4 function as promiscuous multitaskers themselves, interacting with other DAMPs such as the S100 proteins S100A12 (34) and S100A8/A9, respectively (35), ligands such the Aβ amyloid polypeptide and heat shock proteins (HSPs) or PAMPs.

The post-translational redox modifications of HMGB1 with the exception of the sulfonyl-modification are reversible, enabling HMGB1 to switch from its “cytokine task” to its “chemokine task” or vice versa. Terminally oxidized sulfonyl-HMGB1 fully lacks inflammatory activity. Distribution of HMGB1 between its “day-time job” (nucleus) and its “night-time job” (extracellular milieu) is further regulated by acetylation of lysine residues within its nuclear localization sequence (Figure 1). Acetylation favors the cytosolic fraction over nuclear HMGB1 and thus is a prerequisite to redirect HMGB1 into the extracellular space (14, 17, 24, 36). A similar role for alarmin hyper-acetylation has also been shown for the nuclear Y-Box protein-1 (YB-1), which is secreted by non-conventional pathways following acetylation (37).

The extracellular cytokine/chemokine-like activities of HMGB1 thus represent true multitasking in the “second” job of this alarmin/DAMP protein (Figure 1).

## Atypical Chemokines as “Multitaskers”

At first glance, alarmins and chemokines, while both playing important roles in host defense and inflammation, are fundamentally different classes of mediators. As outlined above, alarmins are *bona fide* intracellular effectors that upon abundant rapid release alert the environment about cell stress and danger. In apparent contrast, chemokines (and cytokines in general) are *bona fide* extracellular mediators that typically have no role within the cell. As discussed above, the IL-1-type cytokines IL-33 and IL-37 that have intrinsic nuclear activities are exceptions to this rule. While classical chemokines of the homeostatic sub-class such as CXCL11 or CXCL12 are stored intracellularly under resting conditions, they do not appear to fulfill intracellular functions, except for “awaiting” their secretion. Moreover, classical chemokines of the inflammatory sub-class, with some exceptions, are not intracellularly measurable at rest; their production is tightly regulated. Transcription and translation are induced by inflammatory or stress stimulation only, which is when levels rise from essentially “zero” by several hundred- or thousand-fold. Induction also is typically directly coupled with the secretion of inflammatory chemokines into the extracellular space, where they drive leukocyte migration and/or promote inflammation through binding to their cognate chemokine receptors. When secreted from inflammatory endothelium, chemokines such as CXCL1 are deposited on the endothelial surface to form an haptotactic gradient and function as arrest chemokines (38, 39). However, beyond regulation at induction level, some inflammatory chemokines are pre-stored following translation. For example, CCL2 is stored under the endothelial surface as intraendothelial chemokine to guide lymphocytes across an inflamed endothelial barrier, circumventing the need for surface-deposited chemokines or extraendothelial chemokine gradients (40). Moreover, some chemokines are stored as proforms. This applies to the platelet chemokines connective tissue-activating protein III (CTAP-III/NAP-2/CXCL7) and platelet factor 4 (PF4/CXCL4), which are pre-stored in platelet granules (41). Furthermore, CX_3_CL1 and CXCL16 are translocated to the plasma membrane, where they are “stored” as transmembrane proforms that are “activated” by proteolytic processing. In this case, proteolysis represents an important regulated “induction” step (42). Moreover, fine-tuning of several other chemokines has been described at the post-translational level, e.g., by N-terminal processing (43–45).

Alarmin receptors are as diverse structurally as alarmins themselves, spanning classes such as scavenger receptors and PRRs, ligand-gated channels, single-spanning helix-type transmembrane proteins, or chemokine receptors as a sub-group of G protein-coupled receptors (GPCRs) [for detailed overview see 2, 7, 11, 39, 40]. In contrast, classical chemokines (CKs) are 8–10 kD small proteins that are uniformly defined by an N-terminal cysteine motif and a characteristic β-strand-rich structural core, featuring the so-called chemokine-fold. Forty-nine classical chemokines interact with 18 GPCR-type classical chemokine receptors (CKRs) as well as five atypical chemokine receptors (ACKRs). The chemokine network is characterized by a high degree of promiscuity with numerous chemokines binding to several receptors and certain receptors engaging more than one chemokine ligand. Classical chemokines are divided into CC-, CXC-, CX_3_C-, and C-type sub-classes owing to the positioning of one or two vicinal cysteines at the N-terminal. The receptors are termed correspondingly (46–48). Chemokines form monomers or dimers, but higher-order oligomers also are observed. The receptors also exist as monomers and dimers, but the precise stoichiometry of ligand and receptor oligomeric combinations is not yet fully understood for most chemokine/receptor pairs (49–52). Thus, proteinaceous alarmins and classical chemokines exhibit fundamental structural and functional differences.

However, intriguing overlaps between these categories of mediators have been identified. First and as outlined above, alarmins, once released into the extracellular milieu, and classical chemokines can directly interact to form heterodimers; all-thiol HMGB1 binds to CXCL12 and HMGB1/CXCL12 dimers elicit CXCR4 signaling responses that are different from those triggered by CXCL12 alone (33); CCL5/HNP1 heterocomplexes represent another example of a dimer between a classical chemokine (in this case the CC chemokine CCL5/RANTES) and a prototypical alarmin (in this case the human neutrophilic peptide HNP1) (53). Secondly, the four classical chemokine categories (i.e., the CC-, CXC-, CX_3_C-, and C-type classes) have more recently been amended by a fifth “functional” class of chemokines, called the chemokine-like function (CLF) chemokines or innate chemokines, or atypical chemokines (ACKs), which share significant functional similarities with classical chemokines, i.e., exhibiting chemotactic activity. Here, we use the term ACK in analogy to ACKRs.

ACKs are a structurally diverse class of small 8–25 kD proteins that lack the classifying N-terminal cysteine residues. Some ACKs share gross architectural similarity with classical chemokines such as extended β-pleated sheet structures with juxtaposing α-helices, but do not contain a cognate chemokine-fold (9, 54–56). Despite these structural differences to classical chemokines, ACKs/CLF chemokines bind to CKRs with high affinity and elicit chemotactic cell migration.

A typical ACK would be a constitutively expressed cellular protein with a *bona fide* intracellular function that can be released or secreted upon cell injury or during inflammatory stimulation to act as a chemokine-like mediator in the extracellular milieu, pinpointing significant similarities with proteinaceous alarmins.

The concept of ACK/CLF chemokines dates back to the work of Wakasugi and Schimmel who discovered that proteolytically cleaved fragments of tyrosine amino-acyl-tRNA synthetase (TyrRS) are secreted into the extracellular milieu, where they act as chemokines by binding to the classical chemokine receptor CXCR1 on neutrophils and monocytes (57). Subsequent work demonstrated similar activities for TrpRS, HisRs, and AsnRS, as well as acting through CXCR1, CXCR3, CCR5, and CCR3, respectively (11, 58–60). Moreover, the p43 auxiliary component of the tRNA multisynthetase complex is identical to endothelial monocyte-activating polypeptide-II (EMAP-II), an inflammatory cytokine with anti-angiogenic properties (61). It was further demonstrated that EMAP-II is identical or similar to the C-domain fragment of TyrRS but also certain non-mammalian proteins (62). Together, this indicated that intracellular housekeeping proteins such as amino-acyl-tRNA synthetases (AaaRS) needed for the ribosomal protein biosynthesis machinery have a “second job” in the extracellular environment as cytokines/chemokines. In the following chapter, we will also discuss the ribosomal protein RPS19, which after release serves as a cytokine antagonist in the extracellular space.

Defensins, in particular, β-defensins, are small cysteine-rich cationic proteins abundantly produced by epithelial and endothelial cells that act as pore-forming amphipathic antimicrobial peptides, also have important “second job” functions as chemokine-like factors. Human β-defensin-1 (HBD1), HBD3, and mouse β-defensin-14 (MBD14) bind to and signal through CCR6 on T cells and dendritic cells, while HBD2 and HBD3 as well as MBD4 and MBD14 additionally interact with CCR2. Moreover, HBD3 acts as an alternative ligand for CXCR4 (63–67). These studies also identified an important structural determinant for the promiscuous usage of classical CKRs by non-classical chemotactic mediators. HBD1 and 2 express a positive charge cluster on their surface that is very similar to that of the cognate CCR6 ligand CCL20/MIP-3α, representing a remarkable example of structural mimicry (68). Additional examples of ACK/CLF-type chemokines are thioredoxin (for which the CKR has not yet been identified) (69), the cold shock protein YB-1, which upon secretion not only interacts with Notch-3 but also with CXCR2 (70), as well as cathelicidin-related antimicrobial peptides such as LL-37/Cramp-1, which bind to CKR-like formyl-peptide receptors, although it may be argued that cathelicidins share more similarity with inflammatory neutrophilic cytokines secreted by conventional pathways than with alarmins or ACKs (18, 71–73). Similarly, serum amyloid A (SAA), a prominent liver-derived pro-inflammatory acute phase protein exhibits ACK-like properties owing to its signaling activity through the chemoattractant receptor formyl-peptide receptor-2 (FPR2), although part of its initially described chemotactic activities were more recently redefined as “indirect” effects via TLR2 (74).

In any case, all these ACK-type proteins have typical “moonlighting” characteristics, featuring “night-time (second) jobs” as chemokine-like mediators in inflammation, autoimmunity, and host defense. Also, although they are structurally diverse, some of them share mixed β-sheet/α-helix structural elements that are reminiscent of the β-sheet/α-helix architecture of classical CKs. Table 2 summarizes known ACK, indicating their moonlighting functions and receptors vs. intracellular job 1-interactors.

**Table 2:** List of known atypical chemokines (ACKs), their “hijacked”^*^chemokine receptors, other receptors, and intracellular interaction partners.

**Atypical chemokine (ACK)**	Engaged receptor(s)^******^	**Intracellular binding partner(s) (*if any*)**
MIF	CXCR2, CXCR4, CXCR7 CD74^**^	CSN5/JAB1, peroxiredoxin (PRX), thioredoxin (TRX), p53, AIF, mutSOD1, p115
MIF-2	CXCR4 (?)^***^ CD74^**^	*Unknown*
HMGB1/CXCL12	CXCR4 (via formation of a heterodimer with CXCL12) TLR2/4, RAGE	Chromatin, DNA
Thioredoxin	Unknown^****^	Various redox proteins with active disulfides
**β-Defensins**		
HBD1 HBD2 HBD3 MBD4 MBD14	CCR6 CCR2 CXCR4, CCR6, CCR2 CCR2 CCR6, CCR2	N/A
HNP-1/CCL5	CCR5 (via formation of a heterodimer with CCL5)	N/A
**Aminoacyl-tRNA synthetases (AaaRS)**		
TyrRS EMAP-II/p43-C-domain TrpRS HisRS AsnRS	CXCR1 CXCR1 CXCR1, CXCR3 CCR5 CCR3	Various interactions with the ribosomal protein/rRNA machinery
LL37/Cramp-1	FPR2	N/A
Serum amyoid A (SAA)	FPR2 TLR2	N/A
Viral chemokine mimics^*****^		
HIV gp120 v-MIP	CXCR4, CCR5 CXCR4	N/A

* *The utilization of chemokine receptors by mediators that do not formally belong to the structural class of classical chemokines, is also referred to as “molecular hijacking,” emphasizing the binding between an ACK and a classical chemokine receptor of the CC or CXC subclasses*.

** *CD74 is not a non-chemokine receptor and is often referred to as the cognate MIF receptor*.

*** *While experimental evidence is yet missing, it has been speculated that MIF-2 may interact with CXCR4 but not CXCR2 due to the lack of a pseudo-ELR motif*.

**** *It has been suspected that thioredoxin elicits monocyte chemotaxis via engaging a chemokine receptor, but this receptor has yet remained elusive (69)*.

***** *There are numerous examples of “viral chemokine mimicry” mechanisms, involving mimicry of host chemokine receptors or ligands, e.g., to facilitate viral entry into host immune cells. Here, we only list HIVgp120 and viral MIP (vMIP) as prototypical examples; others are summarized in recent reviews, e.g., (75–79)*.

****** *For other references see main text*.

## MIF Family Proteins are Atypical Chemokines And Designated “Multitaskers”

Work in the last decade has demonstrated that MIF family proteins are prototypical ACKs that feature all characteristics and multitasking properties of these proteins.

Macrophage migration-inhibitory factor (MIF) was identified in 1966 as one of the first cytokines to be discovered (80). Initially described as a T-cell-derived inhibitor of random macrophage migration, MIF has been redefined as a pleiotropic chemokine-like inflammatory cytokine that acts as an upstream mediator of innate and adaptive immunity. This also suggested that the eponymous migration-inhibitory activity measured 50 years ago, probably is some kind of “desensitization effect” of a chemokine-type mediator. Owing to its potent inflammatory activity profile, MIF has been found to be a pivotal mediator of acute and chronic inflammatory diseases such as septic shock, acute respiratory distress syndrome, neuroinflammation, or rheumatoid arthritis. Dysregulation of MIF also drives atherogenesis and other cardiovascular conditions as well as tumorigenesis. Several excellent review articles have summarized the role of MIF and its homolog MIF-2/D-dopachrome tautomerase (D-DT) as inflammatory cytokines and their significance in numerous diseases (39, 81–92).

Here, we will mainly address MIF's CLFs and its role as ACK and alarmin-type mediator. MIF is an evolutionarily conserved protein that is expressed in most species and kingdoms. Its structure is unique and cannot be grouped into any of the known cytokine classes, but despite an only 20–30% sequence homology, MIF proteins show a remarkable architectural similarity to a class of bacterial isomerases/tautomerase (83, 93). MIF also shares with these proteins a conserved catalytic tautomerase pocket that contains an unusually acidic proline residue, but the significance of this activity in mammals is unclear (82, 83, 91). There is also a remote similarity between the three-dimensional structure of CXC chemokines such as CXCL8 and MIF (39, 93, 94). The architecture of the MIF monomer resembles that of the CXCL8 dimer. MIF proteins are semi-constitutively expressed in many cell types. Accordingly, upregulation of MIF in inflammation and other stress conditions is mostly regulated at the level of MIF release rather than transcriptional induction, although remarkable exceptions such as in neonatal cells have been noted (82, 95, 96). In fact, following translation, MIF is localized in the cytosol and does not enter the ER/Golgi pathway. In analogy, to the above-discussed storage of some classical inflammatory chemokines, it could be argued that cytosolic deposition of MIF following its translation represents “cytosolic storage” awaiting secretory signals. In fact, MIF secretion occurs by a non-conventional pathway involving ABCA1 transporters, p115 and JAB1/CSN5, but the mechanistic details are still unclear (95, 97, 98). Despite the non-canonical nature of the MIF secretion process, release of MIF into the extracellular space is tightly controlled by inflammatory and immune stimulation and occurs in a regulated fashion. In addition, it has been suggested that some tumor cells as well as certain endothelial and epithelial cells release low concentrations of MIF in a semi-constitutive -autocrine- manner that would be independent of a specific and acute trigger.

Once released into the interstitial space and/or circulation, MIF functions to modulate the activity of numerous immune and inflammatory cells. When secretion occurs from inflamed endothelium, e.g., in atherogenesis, MIF is immobilized on the endothelial surface to form an haptotactic gradient. Deposition is similar to atherogenic arrest chemokines and involves interaction of basic surface charges of MIF with negatively charged proteoglycans (94). Circulating or immobilized MIF then engages one or more of its receptors, typically expressed on myeloid cells or lymphocytes. This is the surface-expressed form of CD74/invariant-chain, which also acts as an MHC class II chaperone facilitating antigen loading to class II complexes in the endoplasmic-reticulum (99), as well as the CKRs CXCR2 and CXCR4, the cognate receptors for the ELR+ CXC chemokines CXCL1, CXCL2, or CXCL8, and the ELR- CXC chemokine CXCL12/SDF-1α, respectively (94). CD74 may be also expressed in class II-negative cells, i.e., upon inflammatory stimulation, and exhibits a major role as cytokine receptor for MIF and MIF-2, driving proliferative responses (100, 101). CXCR2 is mainly expressed on neutrophils and monocytes/macrophages, but can also be upregulated on a number of other cell types under inflammatory stimulation. CXCR4 is ubiquitously expressed, but expression levels can be elevated in inflammation. High-affinity binding of MIF to CXCR2 and CXCR4 represents non-cognate interactions between an ACK and CKRs (Table 2) and gives rise to leukocyte recruitment responses in inflammation and atherogenesis (39, 86, 92, 94). Binding of MIF to CXCR2 drives atherogenic recruitment of monocytes and neutrophils (94, 102, 103). MIF/CXCR4 binding supports the recruitment of atherogenic T and B lymphocytes (94, 104, 105), but also regulates the egress of metastatic tumor cells and progenitor cell recruitment (39). All three receptors have important roles in inflammation and cardiovascular disease. The surprising AMP kinase-mediated cardioprotective effects of MIF and MIF-2 in myocardial ischemia/reperfusion injury are mediated by cardiac-expressed CD74 (106–108). The binding of MIF-2 to CXCR2 and CXCR4 has not been addressed, while MIF has also been suggested to interact with CXCR7-mediated pathways (109).

The structural determinants underlying the promiscuous binding of MIF to CXCR2 have been elucidated: MIF/CXCR2 binding is similar but not identical to that of the cognate ligand CXCL8 and requires an N-like loop and a pseudo-ELR motif (39, 103, 110, 111). This represents another intriguing example of molecular mimicry that is reminiscent of how the ACKs HBD1 and 2 mimic the receptor binding motif that CCL20 utilizes to engage CCR6 (68). Recent data also inform about the molecular determinants that govern the MIF/CXCR4 interaction. They show that the MIF/CXCR4 interface involves an extended N-like loop of MIF, an RLR motif, and the N-terminal Pro-2 of MIF as well as sequences from two extracellular loops of CXCR4 (112, 113). Overall, the binding motifs controlling MIF/CXCR4 binding appear to be less similar to those of the cognate binding pair CXCL12/CXCR4, thus representing an example of “remote” molecular mimicry (75, 114, 115).

**Figure 2:**
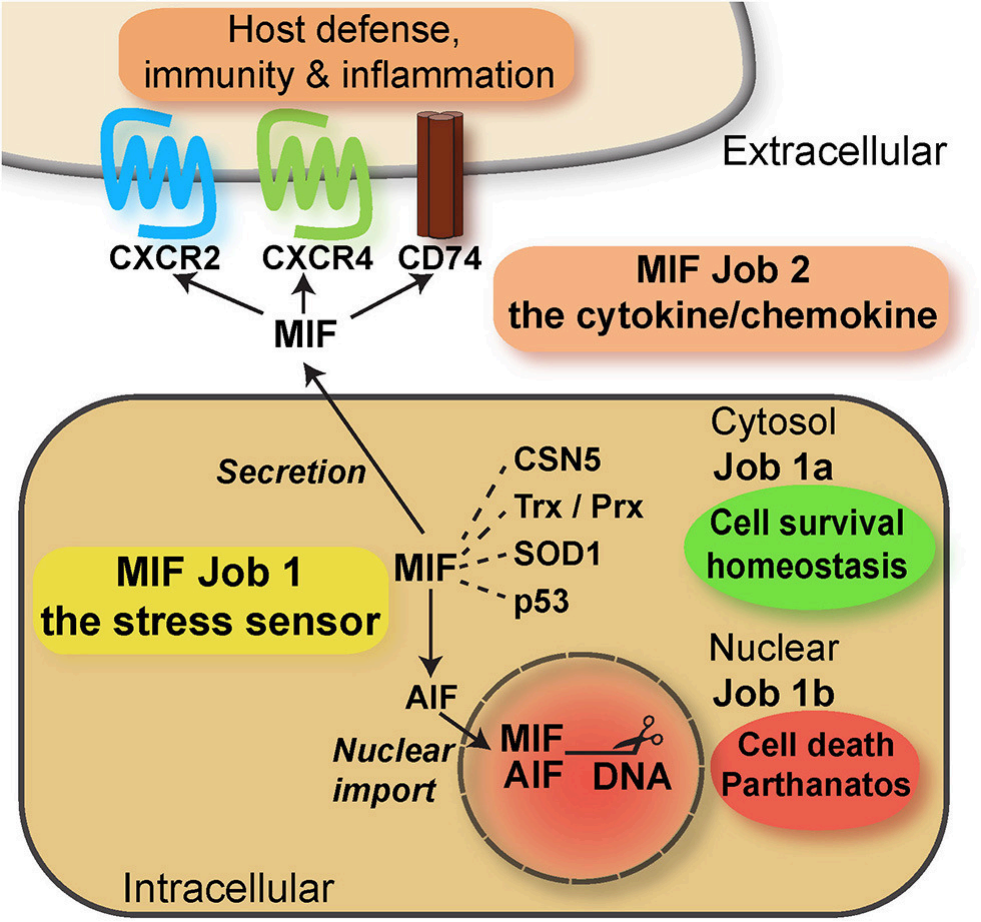
Scheme summarizing the multitasking/moonlighting functions of MIF proteins. For overview purposes, only MIF but not MIF-2/D-DT is depicted. For details see manuscript text. MIF, macrophage migration-inhibitory factor; CSN, COP9 signalosome; Trx, thioredoxin; Prx, peroxiredoxin; SOD, superoxide dismutase; AIF, apoptosis-inducing factor.

In conjunction, these features of MIF proteins are reminiscent of *bona fide* alarmins such as HMGB1 or IL-33, with independent intra- and extracellular roles. In fact, while only speculated about for a long time (116), the potential intracellular activities of MIF proteins have been defined more recently. Intracellular activities were identified both in the cytosolic and nuclear compartment. Cytosolic MIF interacts with CSN5/JAB1 to regulate COP9 signalosome signaling, while inversely, CSN5 is involved in MIF secretion (97, 117). MIF/CSN5 interactions are further linked to apoptotic processes via p53 (97, 118–120). Depending on the oxidative environment, cytosolic MIF also interacts with redox-regulating proteins thioredoxin (Trx) and peroxiredoxin (Prx), and this is mediated by MIF's redox-active cysteine residues (121). While the activity spectrum of MIF's cytosolic interactions is diverse, they may be collectively designated as “cell-protective,” “homeostasis-promoting” activities (Figure 2). This assignment was recently confirmed by an unexpected novel cytosolic interaction partner of MIF. MIF binds to a mutant form of superoxide dismutase (mutSOD1) and inhibits the accumulation of misfolded SOD1, protecting from motoneuron damage (122–124). It is currently unclear whether MIF exerts this function also in non-neuronal cells. The recent discovery of a nuclear function of MIF came as a big surprise. Neurons under ischemic or excitotoxic stress accumulate cytosolic poly-ADP ribose (PAR) trees following DNA damage. This leads to mitochondrial release of apoptosis-inducing factor (AIF), which binds to MIF and escorts it into the nuclear compartment. Nuclear MIF further promotes DNA damage by direct nuclease regulation, leading to cell death by parthanatos (125). While this study has yet to be independently confirmed and while it is unclear whether MIF-2 exhibits similar activities, it supports the concept that MIF proteins can exert their functions in three independent compartments: (i) in the extracellular space, (ii) in the cytosol, and (iii) in the nucleus, representing a intriguing example of “topological multitasking” (Figure 2). With respect to the moonlighting concept, it also means that the intracellular functions (cytosolic and nuclear) would be the intracellular job of MIF (“job 1”), with cytosolic functions designated as “job 1a” and the nuclear activity being “job 1b”. The cytokine/chemokine functions of MIF in the extracellular environment would be MIF's second task (“job 2”) (Figure 2). The activities of MIF may be further fine-tuned by (PTMs), which have been suggested to modulate both intra- and extracellular MIF in a context-dependent manner (Table 1). Interestingly, the intra- and extracellular MIF pools appear to be further linked through other ACK-type/multitasking proteins. MIF and HMGB1 both engage CXCR4 and also are functionally related in a reciprocal manner (33, 94, 126). Moreover, the ribosomal protein RPS19 may be released into the extracellular space, where it binds to and attenuates MIF-mediated inflammatory activities (127).

In conclusion, although the mechanisms regulating the interplay between the different MIF tasks have not yet been elucidated in detail, it is clear that multitasking by MIF over three different topologies and involving several different binding proteins is an impressive example of molecular multitasking, illustrating the functional versatility of MIF proteins and atypical chemokines in general. The ACK characteristics of MIF proteins are also summarized in Table 2.

## Clinical Relevance Of Alarmins And Atypical Chemokines: Can Their Moonlighting Profiles Be Translationally Exploited?

The potential roles of HMGB1 and MIF-family proteins in inflammatory, autoimmune and cardiovascular diseases have been extensively studied and various therapeutic approaches to interfere with their pathologic activities, i.e., antibodies and small molecule inhibitors (SMD) have been explored. This has been summarized in several recent review articles (14, 91, 92, 128–140). Here, we will only briefly allude to the translational opportunities that may arise from specifically targeting the moonlighting characteristics of HMGB1 or MIF proteins.

It seems overall advisable to try to specifically target the extracellular activities of HMGB1 or MIF proteins, i.e., “job 2” and “job 3” (Figures 1, 2). These are the disease-promoting, inflammatory activities of these alarmins/ACKs. HMGB1 enhances immune cell migration (and thus inflammatory recruitment) via job 2 and triggers the release of inflammatory cytokines and chemokines via job 3. MIF, with the exception of its CD74/AMPK-mediated cardioprotective activity in the early phase of cardiac ischemia, amplifies inflammation via various job 2-mediated pathways. These include e.g., cytokine/chemokine upregulation, leukocyte recruitment, and macrophage survival. In contrast, the job 1 activities of HMGB1 and MIF proteins have been suggested to generally be physiologic, maintaining cell homeostasis. Pharmacologic approaches to tackle HMGB1 or MIF should therefore generally preserve their intracellular activities. Antibody-based approaches appear suitable in this respect. Moreover, targeting the receptors appears prudent at first sight, but specificity issues will have to be considered. This is particularly true for MIF receptor-targeted strategies, as the cardioprotective activities of the CD74 pathway and the homeostatic role of CXCR4 should be preserved (92, 141) (Figure 2). Although HMGB1/CXCR4 heterodimers have been found to drive inflammatory leukocyte recruitment (33), this also holds true for CXCR4 blockade strategies as a means to interfere with job 2 HMGB1 pathways (Figure 1).

Additionally, small molecule inhibitors (SMD), directed at the tautomerase site of MIF proteins, represent an interesting strategy to interfere with the pro-inflammatory job 2 MIF activities. However, considering the above-said, those anti-MIF SMDs that are not membrane-penetrable—thus specifically directed at extracellular -job 2- MIFs—overall appear preferable, although exceptions may apply to applications in cancer and stroke, in which intracellular MIF has been suggested to exert detrimental effects as well (125, 131).

In conclusion, while the moonlighting characteristics of alarmins and ACKs constitute complex mechanistic networks that are subject to differential regulation in health and disease, they principally offer intriguing translational opportunities with “job”- and thus disease phase-specific targeting options. Multiple clinical applications are in sight, but also require efforts to further elucidate the multitasking circuits of these proteins.

## Author Contributions

JB conceived the concept and wrote the manuscript. AK co-conceived the concept, edited and improved the manuscript, and drafted the table. OG co-conceived the concept, edited and improved the manuscript, and drafted a figure.

### Conflict of Interest Statement

The authors declare that the research was conducted in the absence of any commercial or financial relationships that could be construed as a potential conflict of interest.
